# Supplementary material for: Structure and dynamics of the E. coli chemotaxis core signaling complex by cryo-electron tomography and molecular simulations
Source: Commun Biol. 2020 Jan 10;3:24. doi: 10.1038/s42003-019-0748-0 (PMC6954272; doi:10.1038/s42003-019-0748-0)
Supplement: Supplementary file 1 — Supplementary Information [file 42003_2019_748_MOESM1_ESM.pdf]

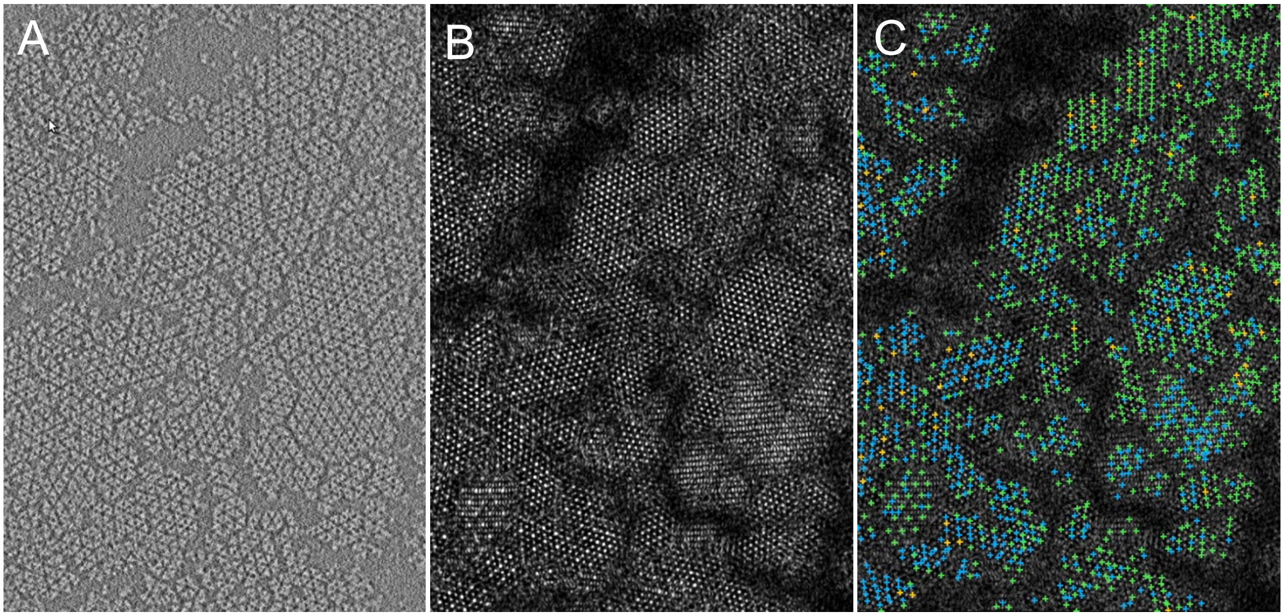

Supplementary Figure 1. Extraction of sub-tomograms by template matching. (A) A slice from a 3D tomogram of the TarCF$_{QEQE}$/CheA/CheW monolayer arrays. (B&C) The corresponding slice of a 3D convolution map obtained by template matching (B) and overlaid with the positions of matched sub-tomograms (C). Colors orange, blue, and green indicate strong-to-weak peak intensities in the convolution map.

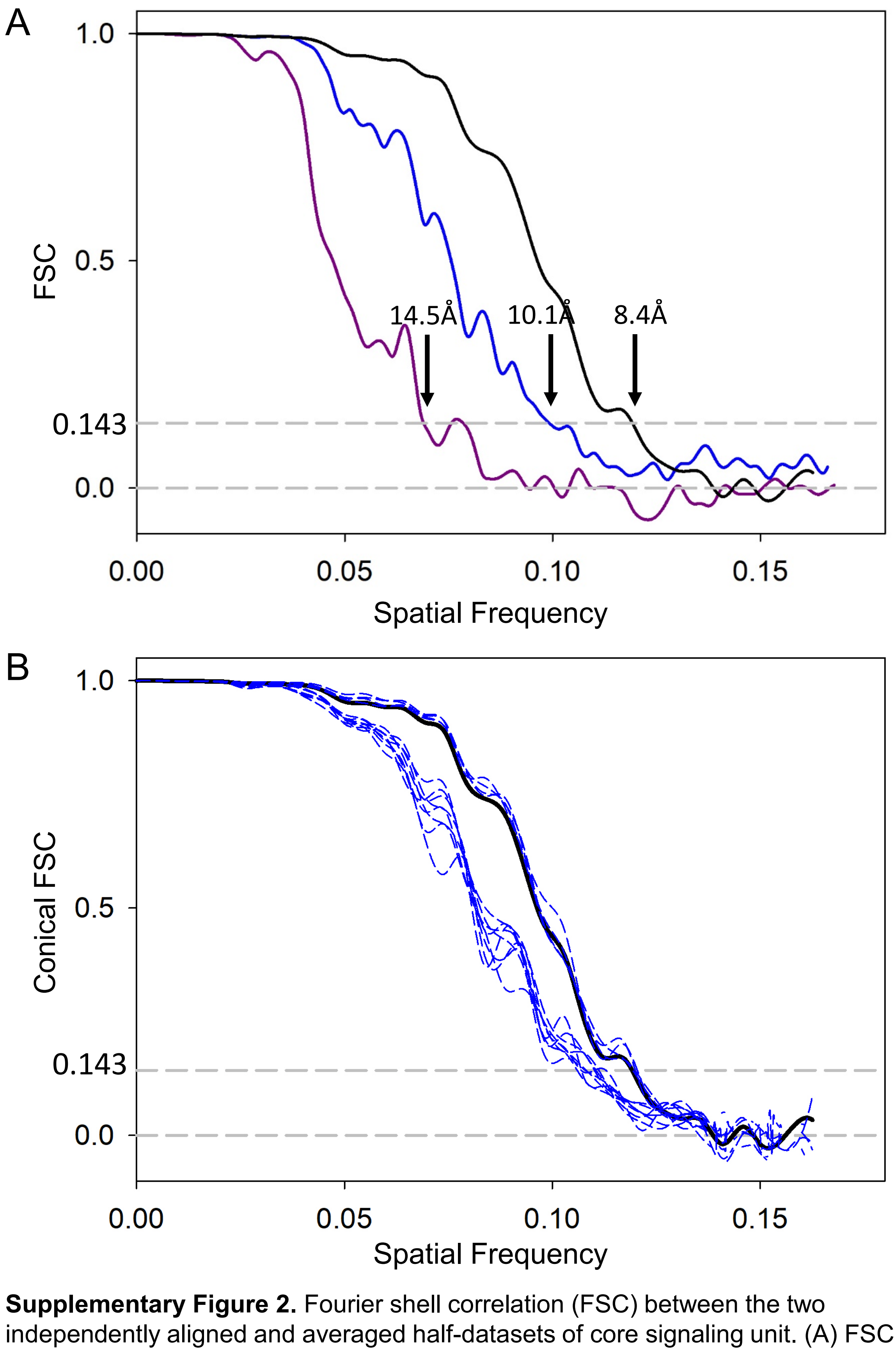

Supplementary Figure 2. Fourier shell correlation (FSC) between the two independently aligned and averaged half-datasets of core signaling unit. (A) FSC plots of sub-tomogram averages of $TarCF_{4E}$/CheA/CheW (Purple), $TarCF_{QEQE}$/CheA/CheW (Blue) and $TarCF_{4Q}$/CheA/CheW (Black). Resolution values are reported at the 0.143 threshold. (B) Conical FSC plots (16 cones, dashed blue) overlaid with the overall FSC (black) of core signaling unit $TarCF_{4Q}$/CheA/CheW.

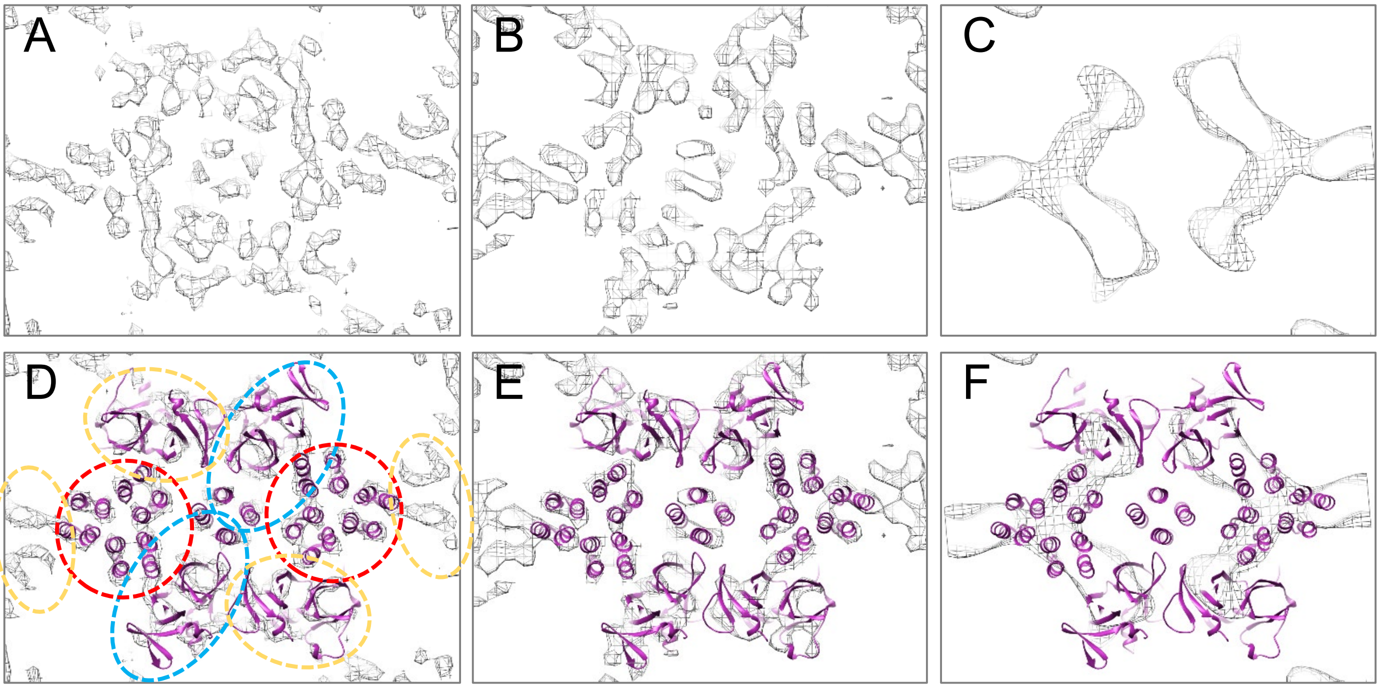

Supplementary Figure 3. CheA.P5 and CheW interact with receptors in a ratio of 1:2. (A-C) Clipped sectional views from Fig. 1 in surface mesh. (D-F) Same sectional views overlaid with atomic models of the core signaling unit. Receptor TODs, CheA.P3.P5, and CheW are marked in (D) with red, blue and yellow dashed circles, respectively.

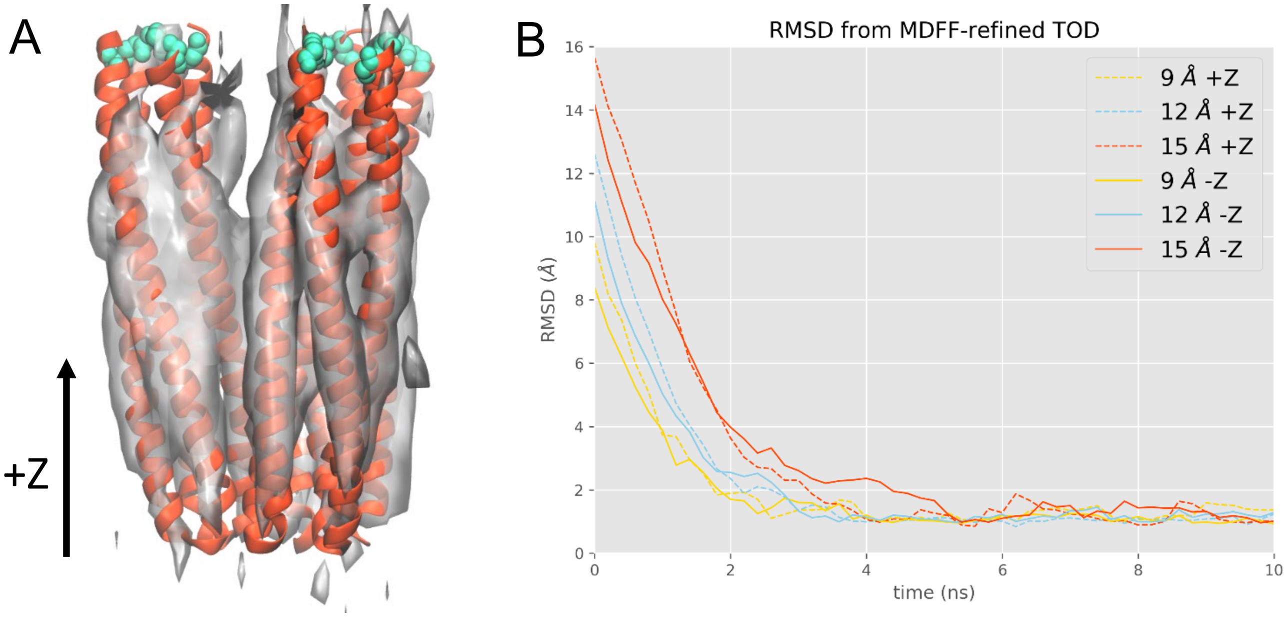

Supplementary Figure 4. MDFF robustly positions receptor trimers-of-dimers (TOD) within 4Q map. (A) Overlay between MDFF-refined Tsr TOD model and corresponding density extracted from 4Q map. Receptors are shown in red; residues comprising the glycine hinge are shown in teal. (B) Timeseries of the root-mean-square deviation (RMSD) between the final frame of the initial TOD MDFF simulation and MDFF simulations of TOD models whose starting positions were shifted in the Z-direction by +/- 9 Å (yellow lines), +/- 12 Å (blue lines), or +/- 15 Å (red lines). See also Supplementary Movie 2.

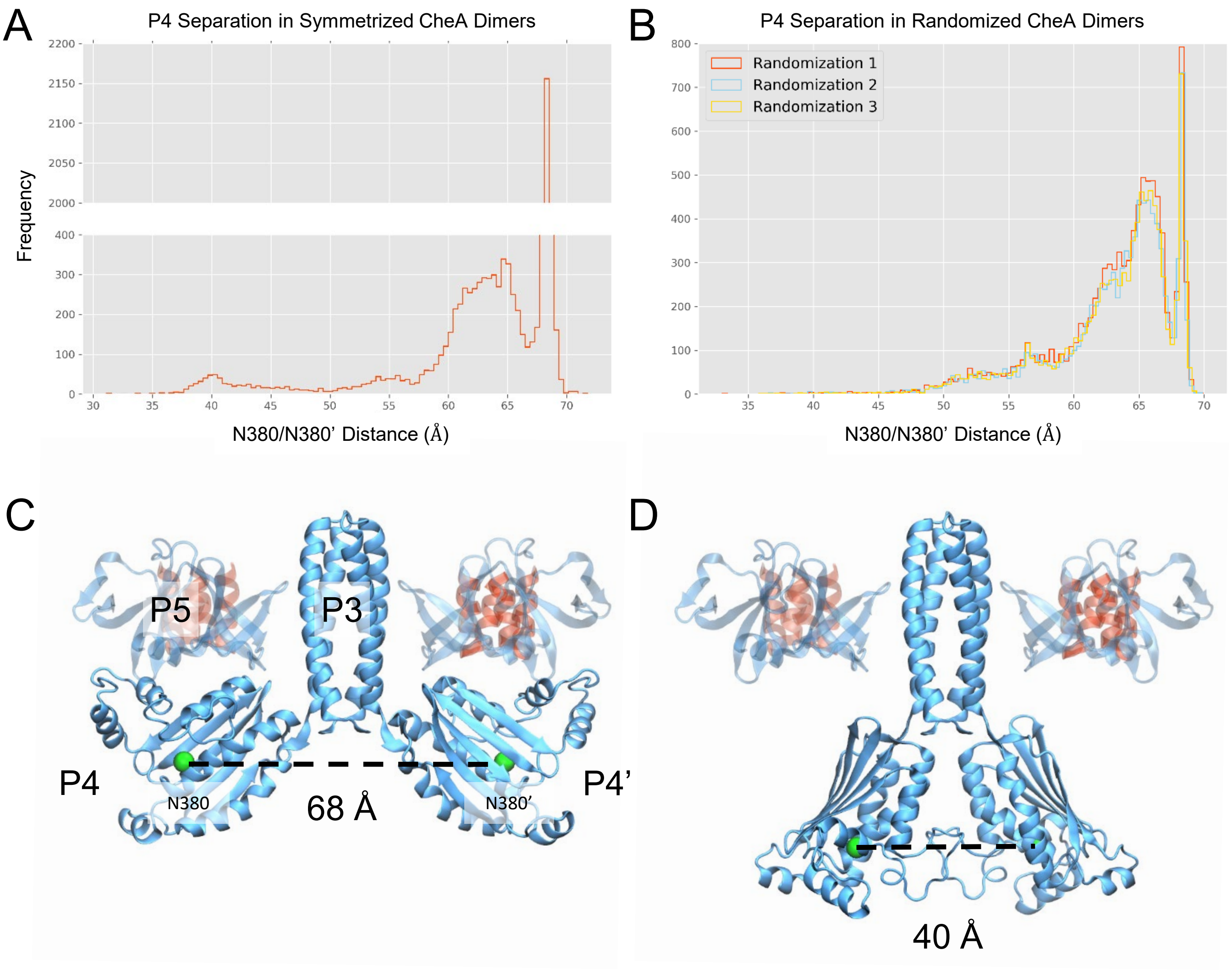

Supplementary Figure 5. CheA.P4 can adopt a wide-range of conformations within the core signaling unit structure. (A-B) Separation between P4 active sites, as measure by the Cβ-Cβ distance of residues N380 and N380', in CheA dimers constructed by combining GSA-derived P4 monomer conformations symmetrically (A) and randomly (B). In (B), each trace corresponds to a different random combination of CheA monomers. (C-D) Representative conformations from symmetrized CheA dimer distribution, showing the P4 domains well-separated (C) and directly interacting (D). CheA.P3.P4 is shown in opaque blue; the Cβ's of residues N380 and N380' are shown as green spheres. For context, CheA.P5 and its bound receptor dimer are shown in transparent blue and red, respectively.

A

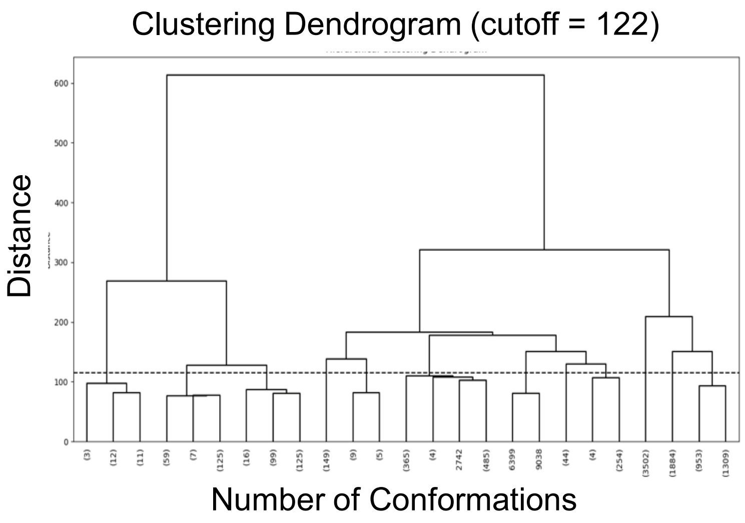

B

Cluster	Number of conformations	% of total ensemble
1	3502	37.15
2	2262	23.99
3	1884	19.99
4	855	9.07
5	258	2.74
6	240	2.55
7	191	2.03
8	149	1.58
9	44	0.47
10	26	0.28
11	14	0.15
12	2	0.02
Cophenetic Correlation Coefficient: 0.892		

C

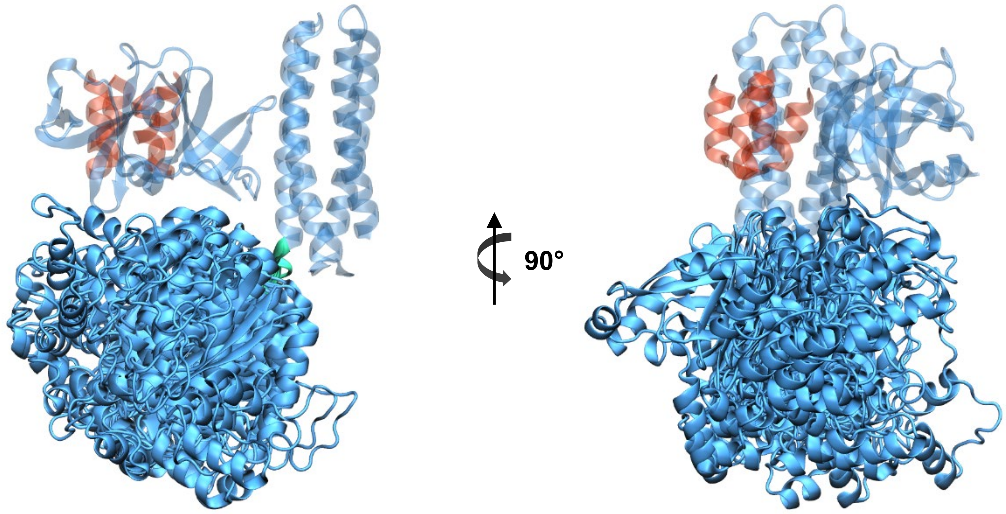

Supplementary Figure 6. Structural clustering of Generalized Simulated Annealing ensemble. (A) Dendrogram resulting from hierarchical clustering of GSA ensemble, truncated at 25 leaves for clarity. A horizontal dashed line denotes the distance cutoff, chosen to be 20% of the maximum distance, resulting in 12 clusters. (B) Number of conformations and percentage of total ensemble represented by each cluster. A cophenetic correlation coefficient > 0.75 suggests the clustering preserves well the original RMSD relationships between conformations. (C) Overlay of 12 representative P4 conformations resulting from clustering analysis. See Methods for more details.

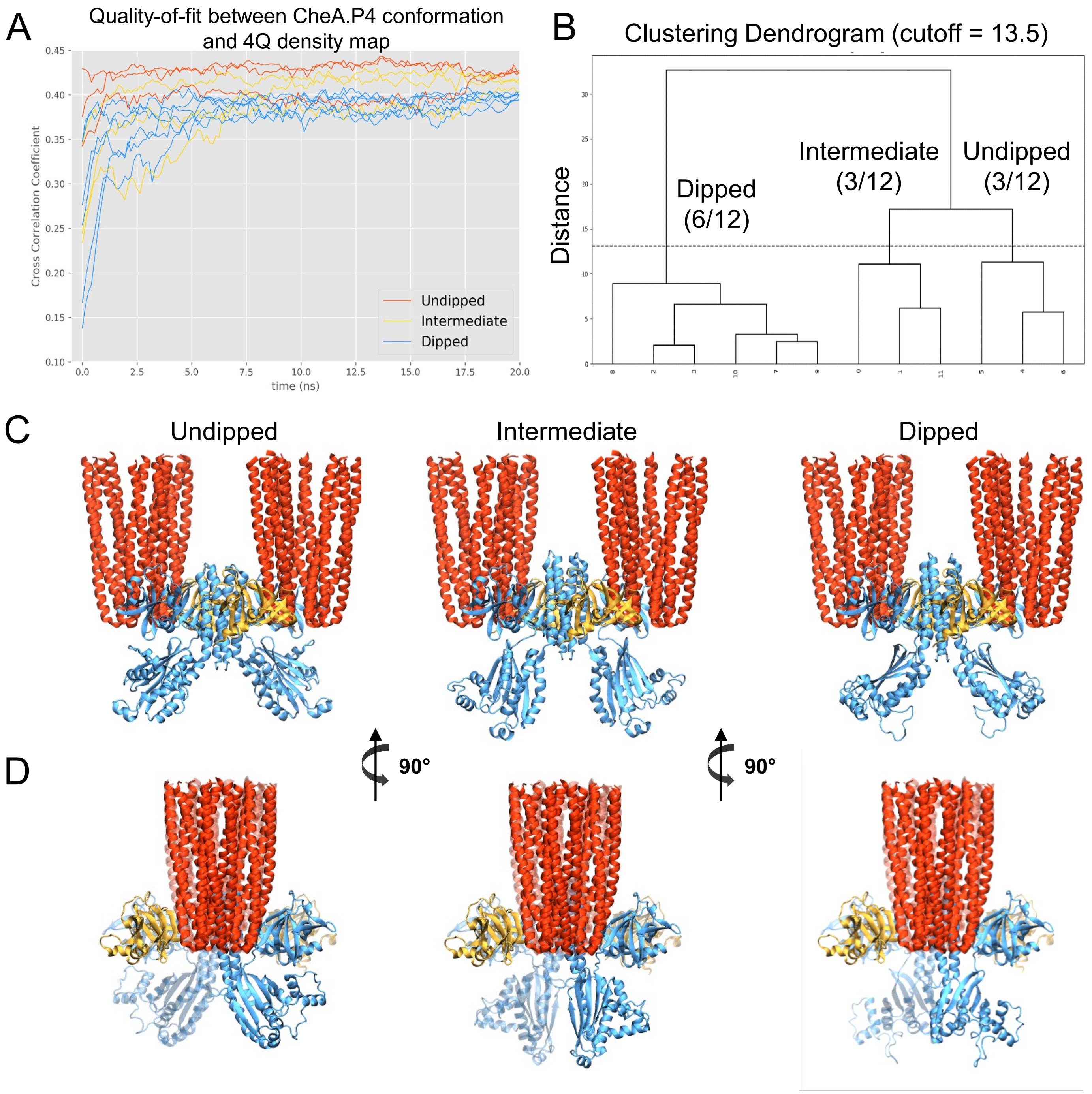

Supplementary Figure 7. 4Q density corresponds to multiple, distinct CheA.P4 conformations. (A) Timeseries of the local cross-correlation coefficient (see Methods) showing the quality-of-fit between each of the 12 GSA-derived CheA.P4 conformations and the 4Q density map plotted over the course of a 20-ns MDFF refinement simulation. Individual traces are colored according to the CheA conformational class to which their final conformation was assigned via hierarchical clustering. (B) Dendrogram resulting from hierarchical clustering of the 12 MDFF-refined CheA conformations. A horizontal dashed line denotes the distance cutoff, chosen to be 40% of the maximum distance, resulting in 3 clusters: “undipped” (three conformations), “intermediate” (three conformations), and “dipped” (six conformations). (C-D) Representative core signaling unit model from each CheA conformational class shown from the front (C) and side (D). The “undipped” and “intermediate” classes are qualitatively similar, but the latter displays an increased P4-P5 separation due to a ~30° increase in the P3-P4 hinge angle.

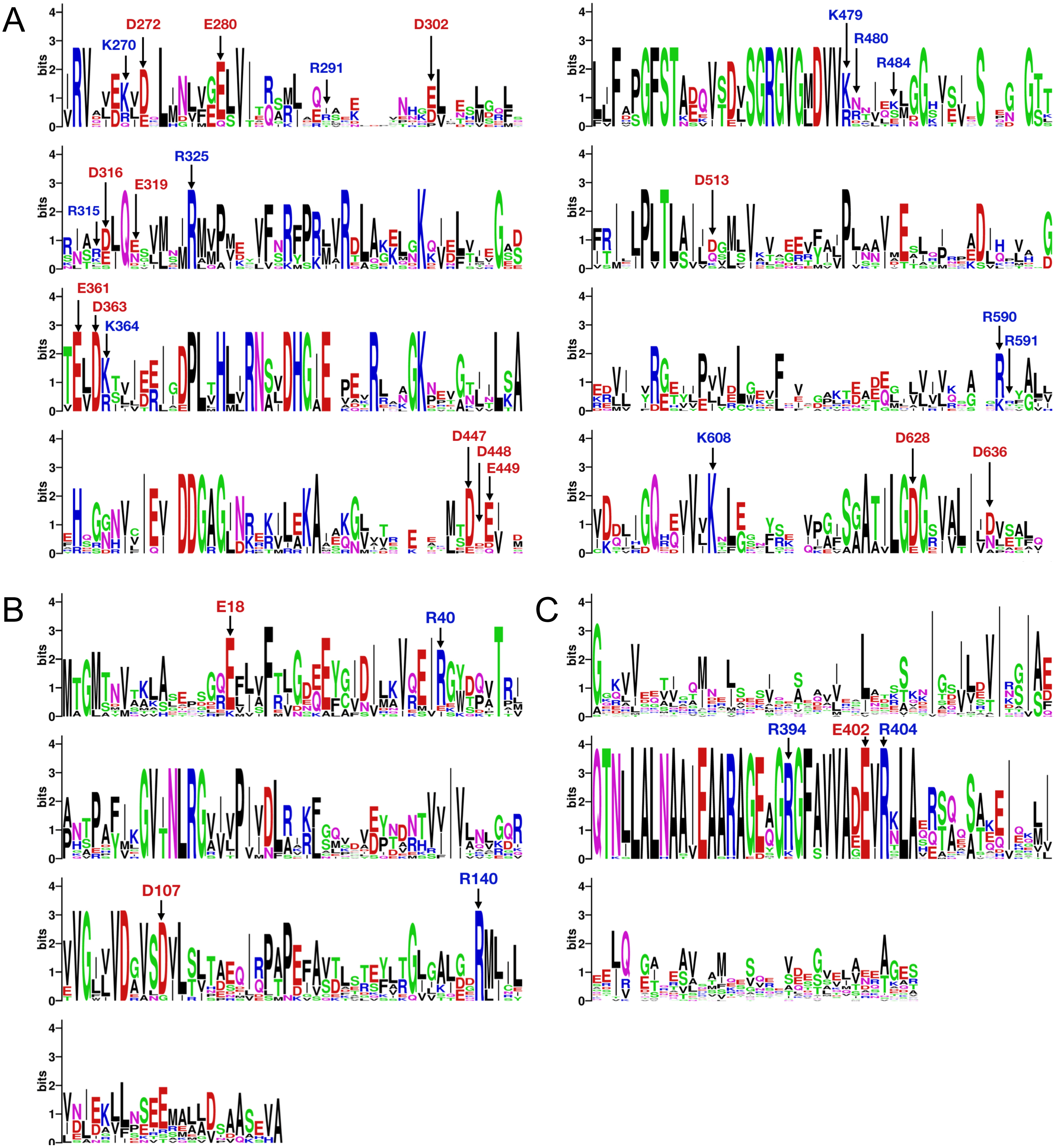

Supplementary Figure 8. Sequence conservation in modeled region of the *E. coli* core signaling unit. (A-C) WebLogos derived from multi-sequence alignments of CheA.P3.P4.P5 (A), CheW (B), and Tsr (C) using https://weblogo.berkeley.edu. Residues discussed in the main text are labeled explicitly. Sequences from a broad range of organisms were obtained for each protein from 'reviewed' datasets on UniProtKB and aligned using Clustal-Omega. Sequences included in the alignments are as follows: CheA [9 sequences/organisms: *ECOLI, BACSU, SALTY, THEMA, KLEAK, RHOSH, LISIN, LISMO, HALS3*], CheW [10 sequences/organisms: *ECOLI, BACSU, SALTY, THEMA, KLEAK, RHOSH, SHIFL, CAUVC, RHIME, RHIEC*], and Tsr [30 sequences/8 organisms: *ECOLI, BACSU, SALTY, THEMA, VIBCH, PSEPK, PSEAE, PSEPU*].

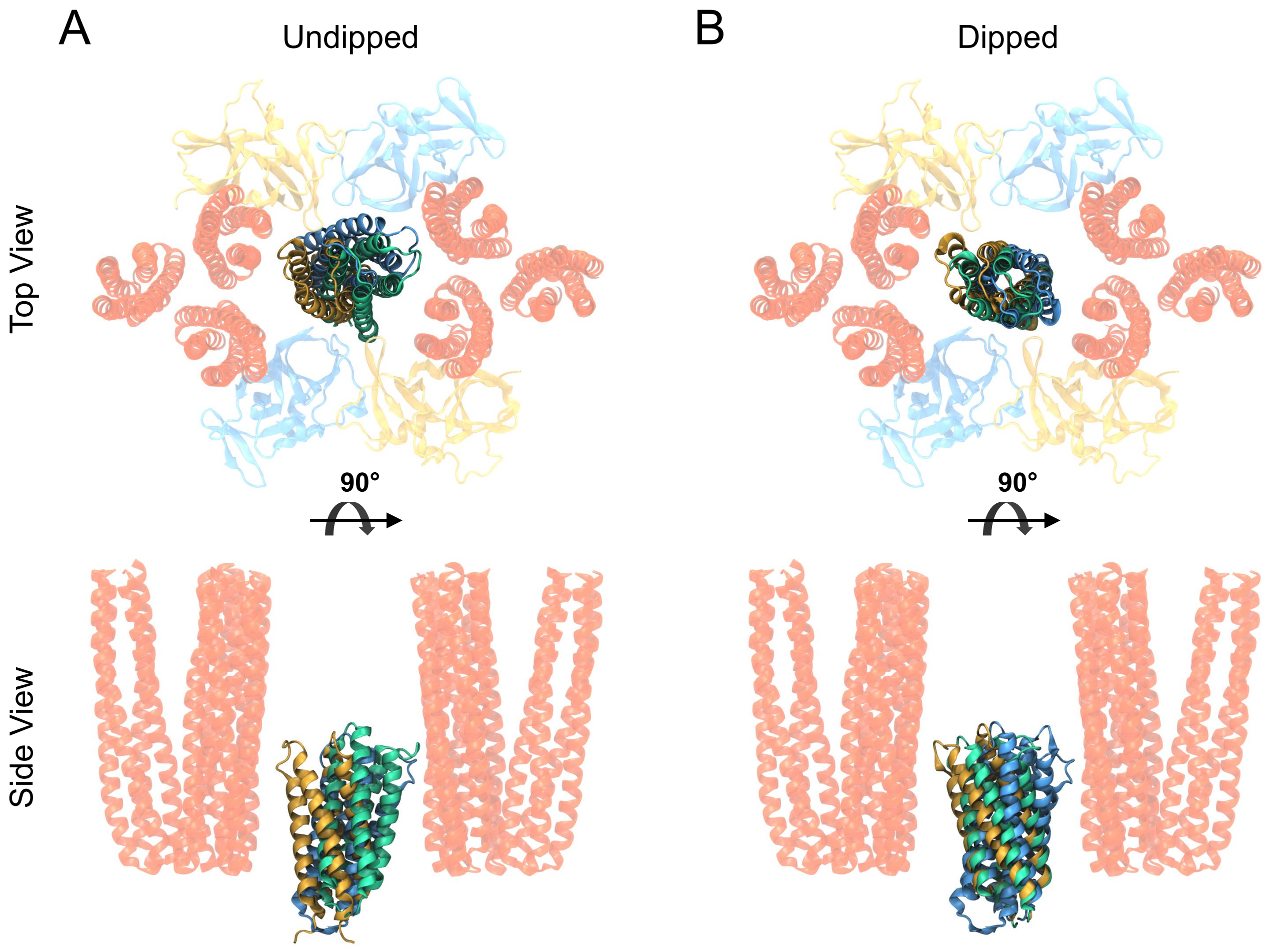

Supplementary Figure 9. CheA.P3 can adopt asymmetric positions within the core signaling unit. (A-B) Final frames taken from three ”undipped” (A) and three “dipped” (B) core signaling unit simulations, showing the position of the CheA.P3 domain from the top and side. Individual P3 four-helix bundles are shown in blue, teal, and gold. For clarity, CheA.P4 is not shown and CheA.P5-CheW are only in the top view.

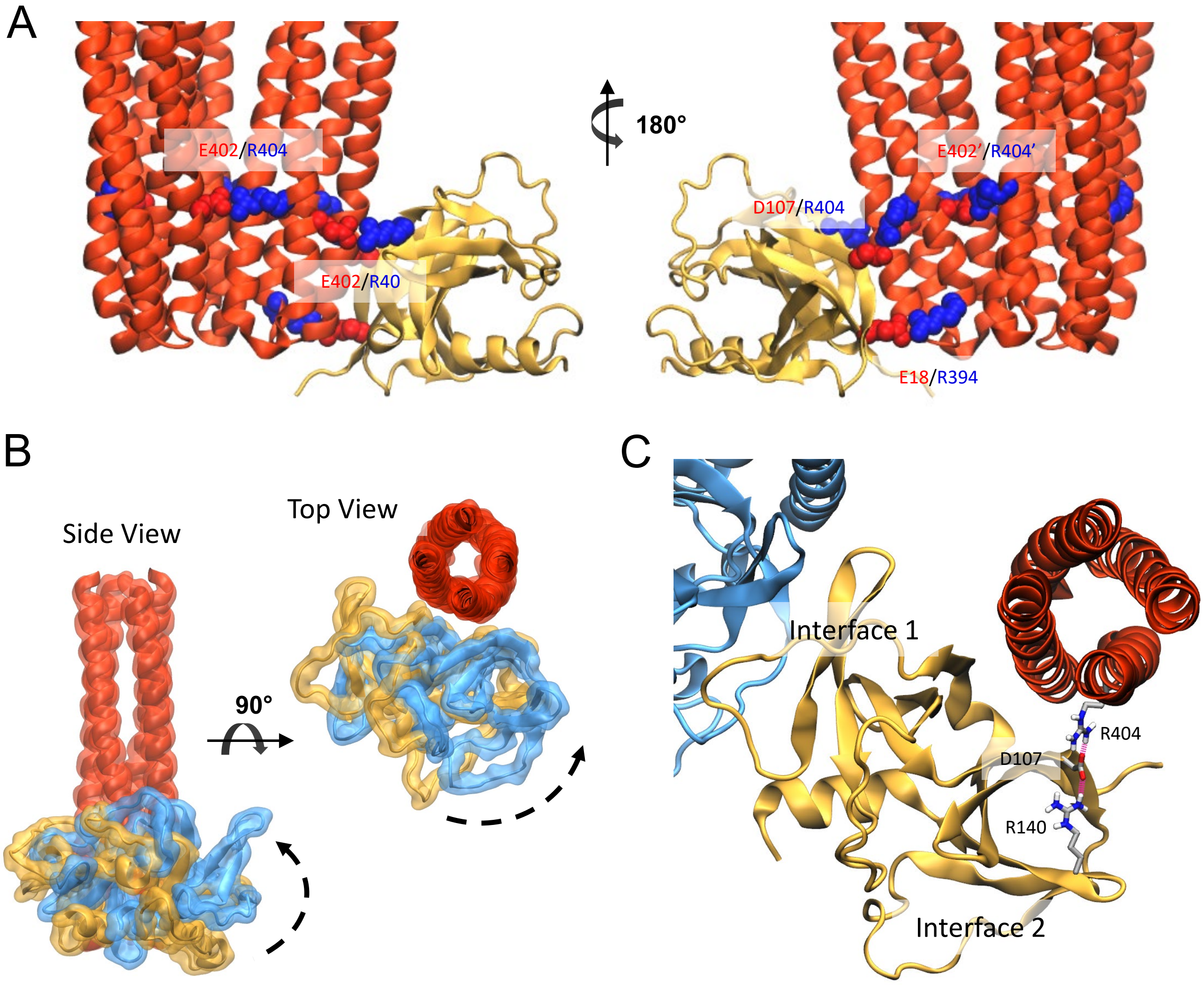

Supplementary Figure 10. Characterization of CheW-receptor and CheA.P5-receptor interfaces. (A) Key salt bridge interactions, involving Tsr-E402 and Tsr-R404, at CheW-receptor and receptor-receptor interfaces. (B) Superposition of the pseudosymmetric CheA.P5-receptor and CheW-receptor interfaces from the side and top. Dashed arrows denote the relative rotation between CheW (gold) and CheA.P5 (blue). (D) Possible allosteric pathway between Tsr-R404 and interface 2, involving salt bridge interactions with CheW residues D107 and R140.

Supplementary Table 1. Summary of cryo-electron tomography data acquisition and image processing.

Acquisition setup			
	Microscope	FEI Polara	
	Voltage (keV)	300	
	Detector	Gatan 4Kx4K CCD	
	Energy-filter	No	
	Å/pixel	2.98	
	Acquisition scheme	Bi-directional	
**Processing**	**4Q**	**QEQE**	**4E**
# of tomograms	24	22	19
Defocus range	-5.0 - 7.0	-4.0 - 6.0	-3.0 - 5.0
Total dose (e^-/Å^2)	60	60	60
Tilt range	-70° to 70°	-70° to 70°	-70° to 70°
# of CSUs	91,636	56,726	33,194
Final resolution in Å	8.38	10.07	14.47

Supplementary Table 2. Summary of protein-protein interfaces observed in MD simulations of the “undipped” and “dipped” CSUs.

Interface	Residues†	Undipped	Dipped	Occupancy
P3-P4	R325-D272	x	x	High in undipped Intermediate in dipped
	R325-D363	x		High
	R325-E361		x	High
	K270-E361	x		Intermediate
	K364-D272	x	x	Low in undipped High in dipped
P4-P5	Cluster 1* (K479,R480,K484)-D636	x		High
	Cluster 2* (D447,D448,E449) -(R590,R591)	x		High
P3-P5	E280-K608	x	x	-
	R315-D513	x	x	-
	R291-D628	x	x	-
P3-R	(D316,E319)-R394	x	x	-
	D302-R409	x	x	-
W-R	R40-E402	x	x	High
	D107-R404	x	x	High
	E18-R394	x	x	Intermediate

†Residue numbers are listed in the order denoted in the “Interface” column. Interactions involving multiple potential partners within the same class are denoted with parentheses. Occupancies computed for the two contact “clusters” at the P4-P5 interface, denoted with a ”*”, consider that at least one of the possible contacts within the cluster is present in a given frame. Due to their asymmetric nature, occupancies for the P3-P5 and P3-R interface contacts are not given.
