# Supplementary material for: Structure and dynamics of the E. coli chemotaxis core signaling complex by cryo-electron tomography and molecular simulations
Source: Commun Biol. 2020 Jan 10;3:24. doi: 10.1038/s42003-019-0748-0 (PMC6954272; doi:10.1038/s42003-019-0748-0)
Supplement: Supplementary file 2 — descriptions of additional supplementary files [file 42003_2019_748_MOESM2_ESM.pdf]

Supplementary Movie 1. Longitudinal slicing through overlay between 4Q (purple) and QEQE (green) density maps.

Supplementary Movie 2. MDFF refinement of Tsr trimer-of-dimers models with starting positions shifted in the Z-direction by +15 Å (left) and -15 Å (right) relative to the initial rigid docking.

Supplementary Movie 3. Longitudinal slicing through overlay between 4Q density map and atomic model of the *E. coli* core signalling unit.

Supplementary Movie 4. Final frame from an "undipped" core signaling unit simulation in which the CheA.P3 domain has adopted an asymmetric position, giving rise to different P4-P5 interactions in each CheA monomer. CheA.P5 and CheW are shown with a transparent representation for clarity.
