# Supplementary material for: Structure and dynamics of the E. coli chemotaxis core signaling complex by cryo-electron tomography and molecular simulations
Source: Commun Biol. 2020 Jan 10;3:24. doi: 10.1038/s42003-019-0748-0 (PMC6954272; doi:10.1038/s42003-019-0748-0)
Supplement: Supplementary file 7 — Reporting Summary [file 42003_2019_748_MOESM7_ESM.pdf]

nature research

Corresponding author(s): Peijun Zhang

Last updated by author(s): Oct 27, 2019

Reporting Summary

Nature Research wishes to improve the reproducibility of the work that we publish. This form provides structure for consistency and transparency in reporting. For further information on Nature Research policies, see Authors & Referees and the Editorial Policy Checklist.

Statistics

For all statistical analyses, confirm that the following items are present in the figure legend, table legend, main text, or Methods section.

n/a	Confirmed	
☒	☐	The exact sample size (*n*) for each experimental group/condition, given as a discrete number and unit of measurement
☒	☐	A statement on whether measurements were taken from distinct samples or whether the same sample was measured repeatedly
☒	☐	The statistical test(s) used AND whether they are one- or two-sided<br>*Only common tests should be described solely by name; describe more complex techniques in the Methods section.*
☒	☐	A description of all covariates tested
☒	☐	A description of any assumptions or corrections, such as tests of normality and adjustment for multiple comparisons
☒	☐	A full description of the statistical parameters including central tendency (e.g. means) or other basic estimates (e.g. regression coefficient) AND variation (e.g. standard deviation) or associated estimates of uncertainty (e.g. confidence intervals)
☒	☐	For null hypothesis testing, the test statistic (e.g. *F*, *t*, *r*) with confidence intervals, effect sizes, degrees of freedom and *P* value noted<br>*Give P values as exact values whenever suitable.*
☒	☐	For Bayesian analysis, information on the choice of priors and Markov chain Monte Carlo settings
☒	☐	For hierarchical and complex designs, identification of the appropriate level for tests and full reporting of outcomes
☒	☐	Estimates of effect sizes (e.g. Cohen's *d*, Pearson's *r*), indicating how they were calculated

Our web collection on statistics for biologists contains articles on many of the points above.

Software and code

Policy information about availability of computer code

Data collection	Cryo-electron tomography data were collected using commercial software FEI-tomo.<br>NAMD v2.13 - parallel CPU/GPU code used for computer simulations
Data analysis	Cryo-electron tomographic volumes were initially reconstructed from tilt series using IMOD, and further processed using emClarity 1.0 (available and referenced in methods).<br>VMD v1.9.4 - molecular visualization program used for basic analysis of computer simulations and rendering of molecular structures<br>python v2.7 - version of python language and libraries used in this study<br>matplotlib v2.2.3 - python module used for creating plot figures<br>scipy v1.2.1 - python module used for performing hierarchical clustering analysis<br>MDAnalysis v0.19.0 - python module used for basic analysis of computer simulations<br>Chimera v1.13 - software used for map segmentation and and rigid-body docking

For manuscripts utilizing custom algorithms or software that are central to the research but not yet described in published literature, software must be made available to editors/reviewers. We strongly encourage code deposition in a community repository (e.g. GitHub). See the Nature Research guidelines for submitting code & software for further information.

Data

Policy information about availability of data

All manuscripts must include a data availability statement. This statement should provide the following information, where applicable:

- Accession codes, unique identifiers, or web links for publicly available datasets
- A list of figures that have associated raw data
- A description of any restrictions on data availability

The cryoET density map of the E. coli 4Q core signaling unit is deposited in EMBD under accession code: EMD-10050. The raw tilt series will be deposited EMPIAR database. Coordinates for the corresponding atomic model are deposited in the RCSB Protein Data Bank under accession code 6S1K.

Field-specific reporting

Please select the one below that is the best fit for your research. If you are not sure, read the appropriate sections before making your selection.

☒ Life sciences ☐ Behavioural & social sciences ☐ Ecological, evolutionary & environmental sciences

For a reference copy of the document with all sections, see nature.com/documents/nr-reporting-summary-flat.pdf

Life sciences study design

All studies must disclose on these points even when the disclosure is negative.

Sample size	For cryoEM structure determination, sample sizes were those required for the resolution. The details of datasets, including sample sizes, are listed in table S1.
Data exclusions	For cryoET subtomogram averaging, standard 3D classification approaches in emClarity were used to exclude data.
Replication	For cryoEM, two randomly divided half datasets were processed independently, and combined to give rise to the final structures. The resolution of the structure is assessed by comparing the two independent maps.
Randomization	cryoEM particles were randomly divided into ODD and EVEN datasets, as standard approach implemented in emClarity.
Blinding	N/A

Reporting for specific materials, systems and methods

We require information from authors about some types of materials, experimental systems and methods used in many studies. Here, indicate whether each material, system or method listed is relevant to your study. If you are not sure if a list item applies to your research, read the appropriate section before selecting a response.

Materials & experimental systems

n/a	Involved in the study
☒	☐ Antibodies
☒	☐ Eukaryotic cell lines
☒	☐ Palaeontology
☒	☐ Animals and other organisms
☒	☐ Human research participants
☒	☐ Clinical data

Methods

n/a	Involved in the study
☒	☐ ChIP-seq
☒	☐ Flow cytometry
☒	☐ MRI-based neuroimaging
